# Supplementary material for: Aging-associated alterations in gene regulatory networks associate with risk, prognosis and response to therapy in lung adenocarcinoma
Source: NPJ Aging. 2025 Jul 9;11(1):61. doi: 10.1038/s41514-025-00247-8 (PMC12241614; doi:10.1038/s41514-025-00247-8)
Supplement: Supplementary file 1 — Supplementary Material [file 41514_2025_247_MOESM1_ESM.pdf]

Aging-associated Alterations in Gene Regulatory Networks Associate with Risk, Prognosis and Response to Therapy in Lung Adenocarcinoma

Supplementary Material

Additional Tables and Figures

Table S.1: Correlation between pathway targeting score of immune pathways and immune score computed by "xcell". Only significant correlations (p-value < 0.05) are reported.

Pathway	correlation	P-value
Primary immunodeficiency	0.171	6.5e-05
T-cell receptor signaling pathway	0.160	1.0e-04
Hematopoietic cell lineage	0.150	4.0e-04
Intestinal immune network for IGA production	0.136	0.002
Allograft rejection	0.136	0.002
Cytokine cytokine receptor interaction	-0.135	0.002
Asthma	0.134	0.002
Autoimmune thyroid disease	0.132	0.002
Type I diabetes mellitus	-0.132	0.002
Graft versus host disease	0.129	0.003
Systemic lupus erythematosus	-0.128	0.003
Natural killer cell mediated cytotoxicity	0.125	0.004
Viral myocarditis	0.119	0.006
Leishmania infection	0.118	0.006
Antigen processing and presentation	0.114	0.008
Chemokine signaling pathway	0.106	0.013

Figure S.1: Aging-associated change in TF-targeting patterns of oncogenes *ERBB3*, *MYCN* and *AKT1* in GTEx. Weights of edges marked in red increase with age and weights of edges marked in blue decrease with age. For each gene top 50 TFs are shown for which the targeting pattern changes most with age.

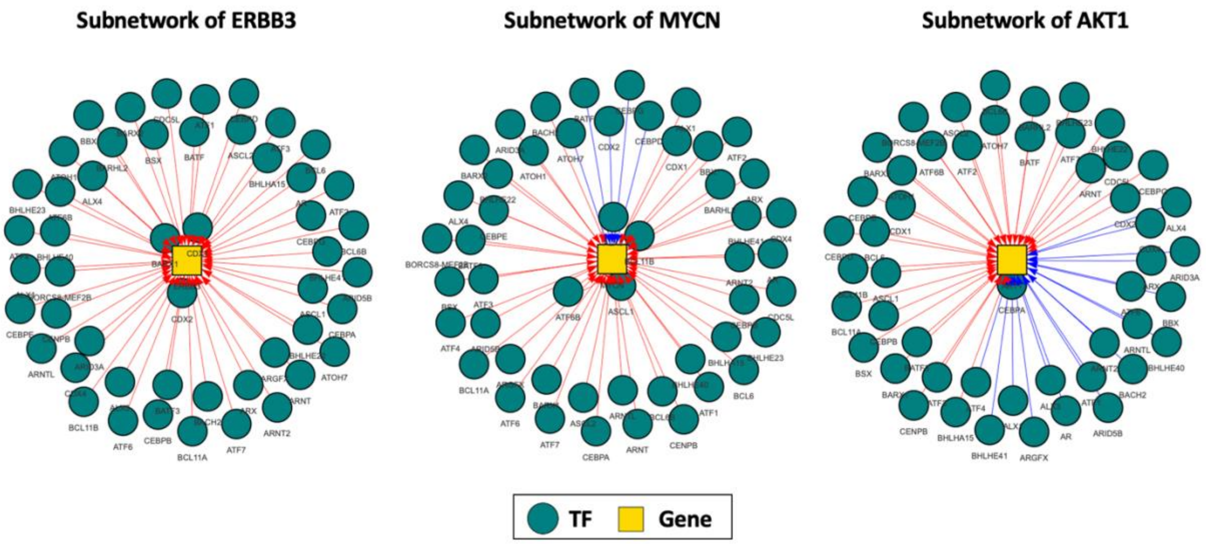

Figure S.2: Aging trajectories for the GTEx samples based on two sets of genes. The plot on the left shows an aging trajectory for smokers (current and past smokers with smoking status = “yes”) and lifelong nonsmokers (smoking status = “No”) constructed based on 1018 genes that are significantly increasingly targeted with age by transcription factors. The plot on the right shows an aging trajectory for smokers (current and past smokers with smoking status = “yes”) and lifelong nonsmokers (smoking status = “No”) constructed based on 404 genes that are significantly decreasingly targeted with age.

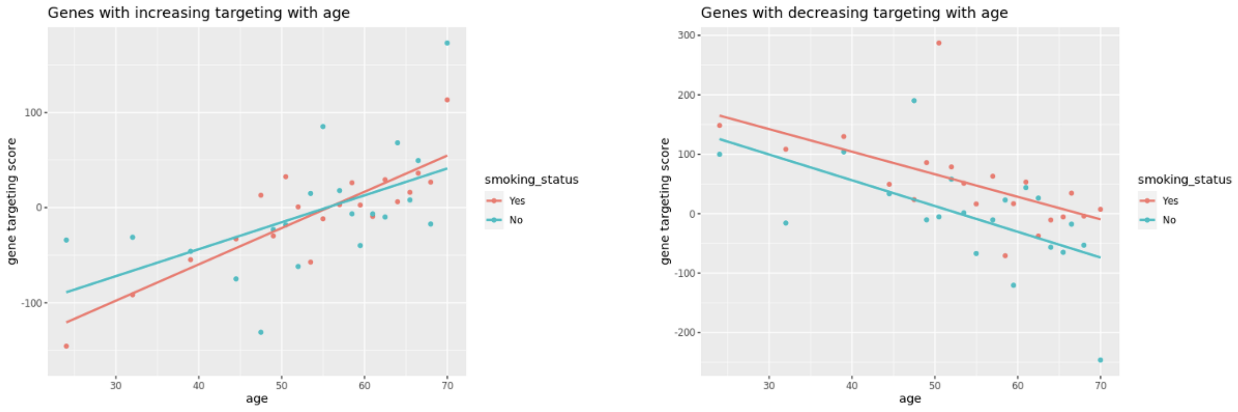

Figure S.3: Aging trajectories for the LGRC samples based on two sets of genes. The plot on the left shows an aging trajectory for smokers (current and past smokers with smoking status = “Ever”) and lifelong

nonsmokers (smoking status = “Never”) constructed based on 888 genes that are significantly increasingly targeted with age by transcription factors. The plot on the right shows an aging trajectory for smokers (current and past smokers with smoking status = “Ever”) and lifelong nonsmokers (smoking status = “Never”) constructed based on 556 genes that are significantly decreasingly targeted with age.

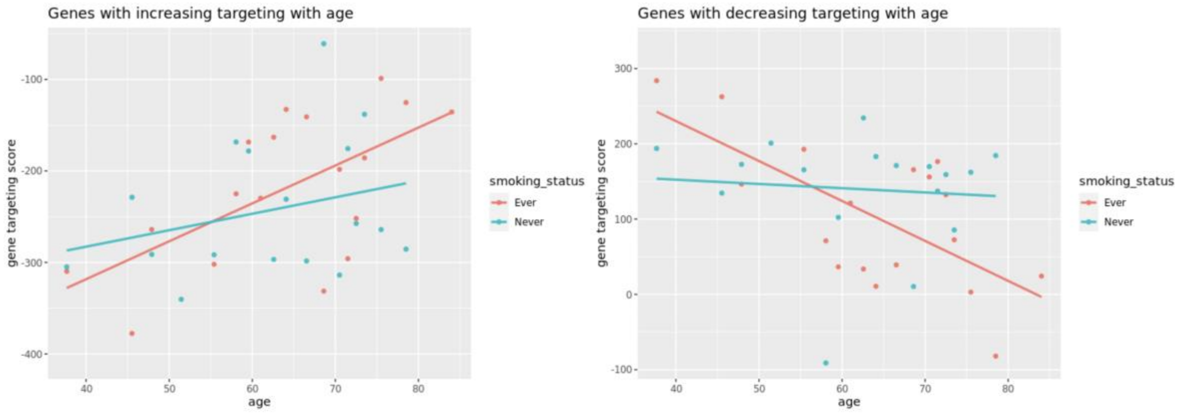

Figure S.4: Change in immune and stromal cell composition with age in GTEx and TCGA: for each cell type, the bar lengths correspond to the t-statistics of the age coefficients from linear models with cell type proportion as response and age as covariate, while adjusting for other clinical covariates. Vertical red dotted lines show the 2.5% and 97.5% quantiles of the standard normal distribution. Cell types for which the corresponding bars cross these lines are inferred to be significantly (p-value < 0.05) changing in composition with age.

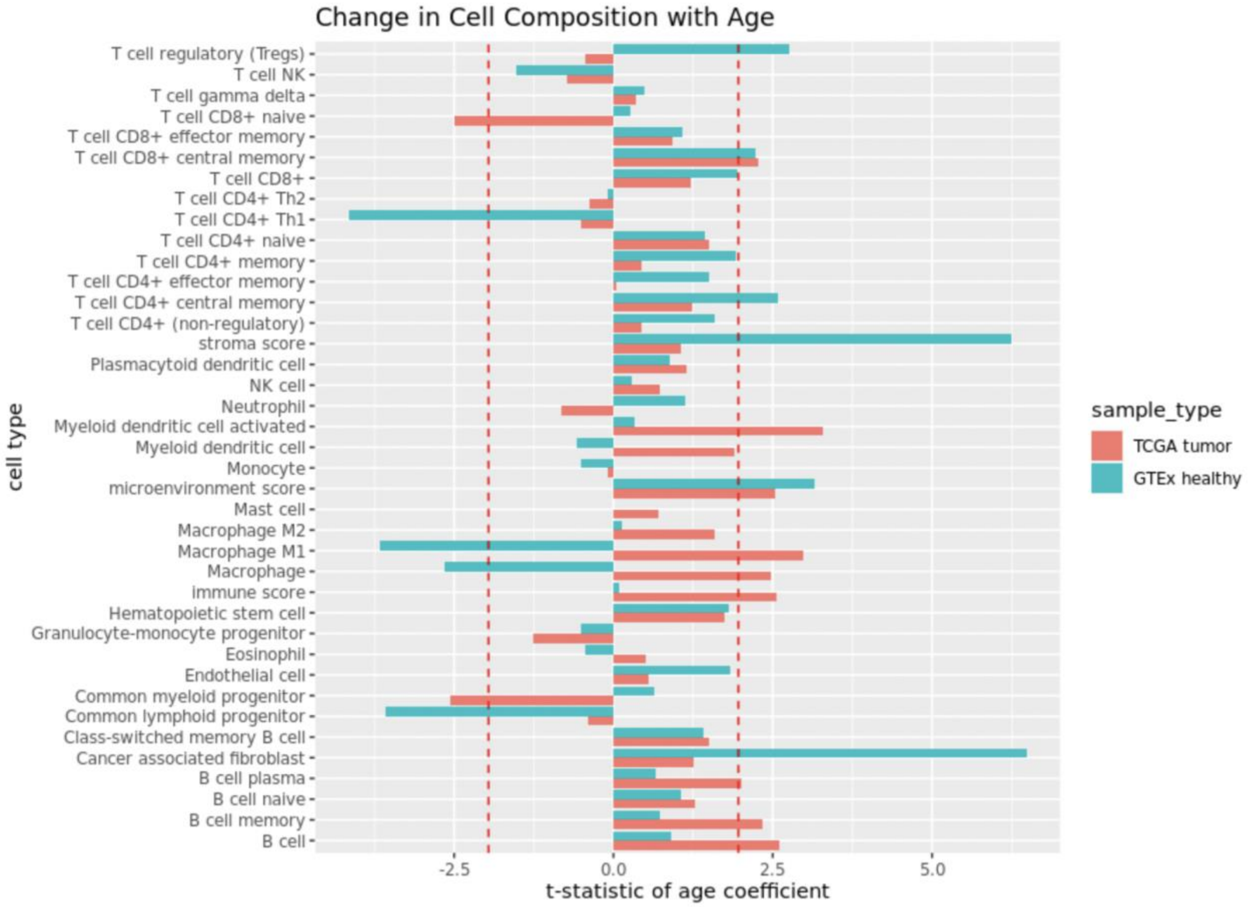

Figure S.5: Kaplan-Meier plot for survival outcome in GSE68465, split by lower and higher network-informed aging signature.

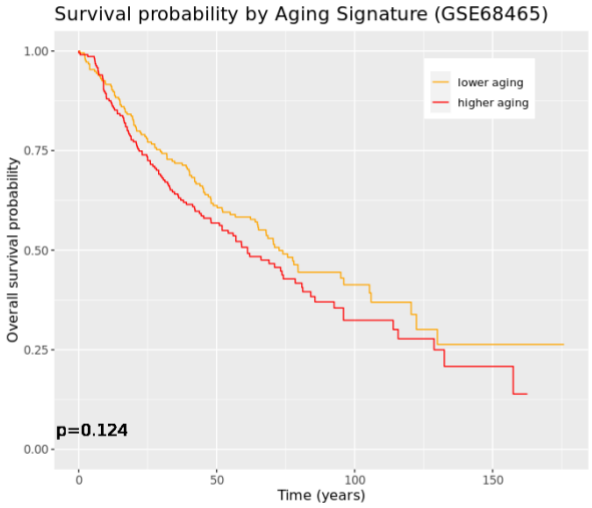

Figure S.6: Venn Diagram of Number of Small Molecule Drug Candidates Derived from CLUEreg as (green) geroprotective drugs, (blue) LUAD drugs for individuals with lower aging signature and (red) LUAD drugs for individuals with higher aging signature.

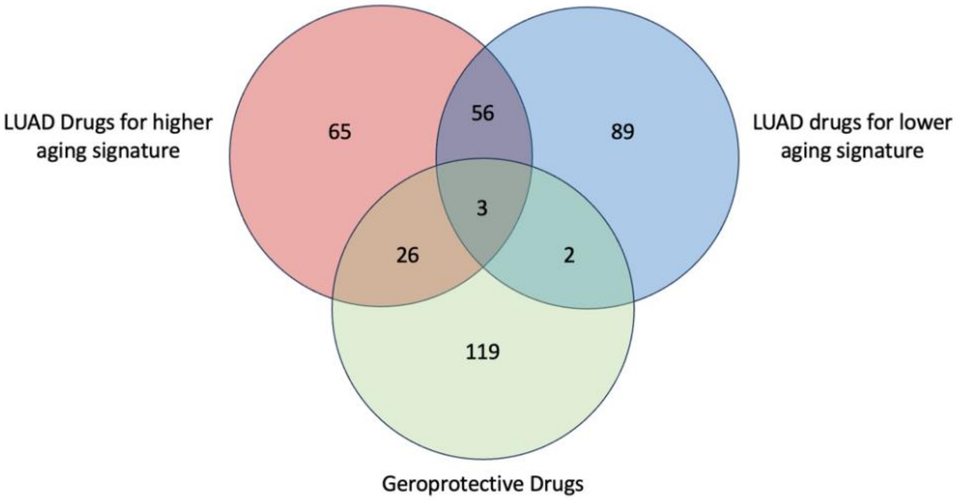

Supplementary Data S1: List of Small-molecule geroprotective drug candidates obtained from CLUEreg
